# Preconception Health and Lifestyle Behaviours of Women Planning a Pregnancy: A Cross-Sectional Study

**DOI:** 10.3390/jcm9061701

**Published:** 2020-06-02

**Authors:** Bonnie R. Chivers, Jacqueline A. Boyle, Adina Y. Lang, Helena J. Teede, Lisa J. Moran, Cheryce L. Harrison

**Affiliations:** 1 Monash Centre for Health Research and Implementation, School of Public Health and Preventive Medicine, Monash University, Clayton VIC 3168, Australia; bonnie.chivers@monash.edu (B.R.C.); Jacqueline.Boyle@monash.edu (J.A.B.); adina.lang@monash.edu (A.Y.L.); Helena.Teede@monash.edu (H.J.T.); lisa.moran@monash.edu (L.J.M.); 2 Department of Diabetes and Vascular Medicine, Monash Health, Clayton VIC 3168, Australia

**Keywords:** preconception, health behaviours, pregnancy planning, women’s health, clinical care guidelines

## Abstract

Preconception care and lifestyle behaviours significantly influence health outcomes of women and future generations. A cross-sectional survey of Australian women in preconception, stratified by pregnancy planning stage (active planners (currently trying to conceive) vs. non-active planners (pregnancy planned within 1–5 years)), assessed health behaviours and their alignment to preconception care guidelines. Overall, 294 women with a mean (SD) age of 30.7 (4.3) years were recruited and 38.9% were overweight or obese. Approximately half of women (54.4%) reported weight gain within the previous 12 months, of which 69.5% gained ≥ 3kg. The vast majority of women (90.2%) were unaware of reproductive life plans, and 16.8% over the age of 25 had not undertaken cervical screening. Of active planners (n = 121), 47.1% had sought medical/health advice in preparation for pregnancy and 81.0% had commenced supplementation with folic acid, iodine or a preconception multivitamin. High-risk lifestyle behaviours including cigarette smoking (7.3%), consumption of alcohol (85.3%) and excessive alcohol consumption within three months (56.3%), were frequently reported in women who were actively trying to conceive. Results indicate that women who are actively planning a pregnancy require support to optimise health and lifestyle in preparation for pregnancy to improve alignment with current preconception care recommendations.

## 1. Introduction

The health status and behaviours of prospective parents before conception, known as the preconception period, is important for the health of women and future generations [1,2]. Initiating strategies to optimise health and lifestyle behaviours during preconception is vital, as some of the most important mechanisms for development and factors affecting birth outcomes occur in the very early stages of pregnancy, often before women are aware they are pregnant or commence antenatal care [3,4]. There are many modifiable health behaviours that can negatively impact outcomes during pregnancy that are difficult to change in the short term [5], and, therefore, addressing these warrants early intervention, during preconception. These include a balanced diet and regular moderate-intensity exercise consistent with recommendations [6,7], weight management, cessation of cigarette smoking, alcohol and recreational drug use, and treatment of sexually transmitted infections (STIs). All of these may also optimise fertility, thereby increasing the likelihood of natural conception [2,5].

The Royal Australian College of General Practitioners (RACGP) Guidelines for Preventative Activities in General Practice [8] recommend a range of preconception care (PCC) strategies, consistent with several other international PCC guidelines [9,10]. Major components of PCC include risk assessment (12), education and health promotion, and medical and psychosocial interventions [11] to enhance knowledge, attitudes and behaviours and improve the health status of prospective parents [10,12]. Understanding whether women seek PCC, what areas of PCC are addressed, as well as their health behaviours during this time, is therefore important, to identify deviation between individual health behaviours and PCC recommendations. This, in turn, can inform and refine PCC recommendations, focus policy and enable targeted PCC strategies where required.

While information is available about the uptake of antenatal care by Australian women [13], little is known about behaviour before pregnancy. Of the available evidence, the majority are retrospective studies of pregnant women [5,14,15]. These studies preclude insight from women who may be planning a pregnancy but do not conceive and are also susceptible to recall bias [16,17,18]. Further, the majority of research relates to a limited number of health behaviours, predominantly folic acid supplementation, alcohol consumption and cigarette smoking [5,19,20]. A comprehensive and holistic understanding of preconception health and behaviours in women currently planning a pregnancy is lacking. To address these fundamental gaps, this cross-sectional study aims to examine health behaviours of women during preconception, in accordance with Australian PCC recommendations [8]. The secondary aim is to compare the results of women at different stages of family planning, to evaluate any differences in behaviour patterns.

## 2. Materials and Methods

### 2.1. Study Design

A cross-sectional questionnaire completed by Australian women in preconception or interconception (between pregnancies).

### 2.2. Health Setting, Recruitment and Participants

The Australian healthcare system is government-supported via ‘Medicare’, which provides universal, free or low-cost care to Australian citizens and residents (and others eligible) across most health services. Private health insurance, paid by the individual, enables choice of hospital and/or provider outside of the public system. Insurance is typically characterised by waiting periods for hospital cover, including pregnancy and birth cover with a 12-month average waiting period before some, or all, of the cost of hospital treatment as a patient is covered. Women of reproductive age who have private health insurance pregnancy therefore provide a unique cohort through which to explore preconception health, before conception actually occurs.

In this study, women were recruited via partnership with a large Australian private healthcare insurance provider, Medibank Private Limited (MPL). Eligible women included those aged between 18–40 years, who had joined or upgraded their health insurance in the previous 12 months to include pregnancy and birth cover, were not pregnant at the time of completing the questionnaire and indicated they were planning a pregnancy within the next five years. Those who indicated they were planning a pregnancy beyond five years into the future, were unsure of, or not, planning a pregnancy or had completed their family were excluded.

A co-developed invitation for participation was emailed by MPL to all eligible women explaining the study and the voluntary nature of participation. Women opted in to the study, providing implied consent, by following an electronic link and completing the anonymous online questionnaire.

### 2.3. Ethics

Ethics approval for this study was obtained by the Monash Health (RES-17-0000-087A) and Monash University (Project no. 10370) Human Research Ethics Committees.

### 2.4. The Questionnaire

The questionnaire was adapted from existing tools to assess women’s pregnancy planning and related health behaviours, risk perception and knowledge. Detailed information about this questionnaire has been published previously [21] and the questions analysed in the current study are provided as supplementary material. It was developed in consultation with multidisciplinary health and medical expertise across obstetrics, public health, social science, dietetics, exercise physiology and endocrinology, with cognitive interviewing and consumer testing performed as previously reported [15]. Information gathered within the questionnaire was aligned with Australian PCC recommendations [8]. This included awareness of a reproductive life plan (a personalised set of goals about pregnancy intention, timing and spacing of intended pregnancies, and associated physical, mental and sexual health considerations [22]); reproductive history; genetic history and screening for genetic conditions; general physical assessment (BMI, cervical screening, STI screening and dental examination history); screening for infectious diseases/immunisation status; folic acid and iodine supplementation; nutrition and exercise; psychosocial factors and substance use (tobacco, alcohol and recreational drugs) [8,23]. General physical assessment, including cervical and STI screening, were analysed in accordance with current Australian guidelines which recommend cervical screening every five years from 2017 for women aged 25–74 years of age that are sexually active (or after two years if last test was prior to 2017, followed by every five years if results are normal) [24]. STI screening recommendations include opportunistic screening of sexually active women under 29 years of age and annual screening for high-risk groups, such as Aboriginal and Torres Strait Islander people, sex workers and women who inject drugs [8].

### 2.5. Stage of Pregnancy Planning

To establish stage of pregnancy planning, women were asked ‘are you planning a pregnancy in the future?’. Women who selected ‘yes, I am currently trying to conceive’ or ‘yes, within 1 year’ were classified as ‘active planners’. Those who answered ‘yes, within 5 years’ were classified as ‘non-active planners’.

### 2.6. Demographics

Socio-economic status was estimated according to participant’s post code, using the deciles in the Australian Socio-Economic Indexes for Areas (SEIFA) Index of Relative Socio-economic Disadvantage [25]. Deciles 1–3 were classified as higher-level disadvantage, 4–7 as moderate-level disadvantage and decile 8–10 as lower-level disadvantage. Rural/remote or urban locality was determined by post code using the Rural and Remote Postcode List [26].

### 2.7. Reproductive Health, Family Planning and Genetic Health

Women were asked what form of contraception was used ‘every time’, ‘most of the time’, ‘sometimes’ or ‘never’ in the last six months when engaging in sexual intercourse. Women could select multiple types of contraception and could select multiple frequencies of use. Selections of ‘every time’ and/or ‘most of the time’ were classified as regular use, and ‘sometimes’ and ‘never’ were captured as non-regular use. Previous and current use of fertility treatment was assessed by the question, ‘have you or your partner been treated for, or are currently undergoing treatment for infertility?’, (yes/no).

### 2.8. Actions to Prepare for Pregnancy

Women were asked if they were currently taking actions to improve their health in preparation for pregnancy, including the following: taking folic acid, iodine, pre-pregnancy multivitamins (analysed individually and combined as a composite) or vitamin D; trying to cut down or stop smoking, cut down or stop drinking alcohol, improve diet, improve exercise level or improve sleep patterns; seeking medical/health advice; not doing any action listed; or taking some other action.

### 2.9. Lifestyle Behaviours and Modifiable Risk Factors

Current and/or recent behaviour relating to alcohol consumption, recreational drug use and smoking was collected. Women were asked to record their average alcohol consumption from Monday–Thursday and Friday–Sunday, per week, for the previous three months, which was collated into a weekly average. The women were also asked to provide the number of times they had consumed more than four standard drinks in one single occasion (excessive drinking) in the past three months: ‘I don’t remember/I don’t know’; or ‘I had stopped drinking alcohol because I was trying to get pregnant’ were provided as additional response options. The cohort were asked if they had ever taken recreational drugs; those who answered ‘yes’ were then asked when the last time they took recreational drugs was. Smoking status was recorded by asking ‘are you currently smoking’, (yes/no). Participants could choose not to respond to questions regarding cigarette smoking or drug use by selecting ‘prefer not to answer’.

Self-reported weight and height were collected and used to calculate BMI (weight/height (m^2^)), which was classified according to the World Health Organization definitions: underweight (≤ 18.49 kg/m^2^); normal weight (18.50–24.99 kg/m^2^) (herein referred to as healthy BMI); overweight (25.00–29.99 kg/m^2^); and obese (≥ 30.00kg/m^2^) [27]. Weight-related behaviours were evaluated, including self-weighing behaviour (daily, weekly, monthly (regular weigher) or occasionally, never (non-regular weigher)), weight maintenance behaviours (maintain or lose weight), and weight gain in the previous 12 months (yes, no or unsure; if yes, how much weight gain: 1–2kg, 3–5kg or 6 + kg).

### 2.10. Statistical Analysis

Data analysis was performed using IBM SPSS Statistics version 25 (Armonk, New York, NY, USA). Descriptive statistics were tested for skewness by using the Shapiro–Wilk test and were presented as mean and standard deviation (SD) for normally distributed continuous variables, and median and interquartile range (IQR) for skewed continuous variables. Frequencies and percentages were presented for categorical variables. The Kruskal–Wallis Test, Mann–Whitney U and the chi-squared test (χ^2^ tests) were used to compare the characteristics of women, stratified by pregnancy intention. A subanalysis was performed for fertility treatment, cervical screening, weight-related actions, weight gain within the previous 12 months and amount of weight gain. To perform subanalyses variables were stratified to explore the characteristics of those within the cohort who reported a response of interest. All *p*-values presented are two-tailed; *p* < 0.05 was considered statistically significant. Where a significant p-value was identified in a multiple comparison, the Bonferroni correction was used to examine if the significance remained after adjusting for multiple groups, and, if so, where significance occurred [28].

## 3. Results

### 3.1. Demographic Characteristics

In total, 4870 eligible women were invited to participate (2104 did not open the original email and were therefore treated as not contactable); of those who opened the email, 23.8% opened the questionnaire and 18.2% (n = 504) attempted it. Ninety-two women reported being currently pregnant and 118 selected a pregnancy intention that did not meet the inclusion criteria, leaving 294 women who met the inclusion criteria for the current study. Overall, the mean age was 30.7 (4.3) years and median BMI was 23.7 (20.1, 26.8) kg/m2. Overall, 41% (n = 121) of women were classified as actively planning for pregnancy while 59% (n = 173) were not actively planning for pregnancy. Women who were actively planning for pregnancy were more likely to be married/de facto (97.8% vs. 90.7%, *p* = 0.04) compared with non-active planners, with no further significant differences in demographic characteristics found between the two groups (Table 1).

Compared with key demographic characteristics from available 2016 Australian Census information [29] across ~3.7 million women aged 18–40 years; 68.9% of our cohort reported a higher annual household income than the population median (~$74,446 AUD/year). The frequency of those reporting unemployment (6.1%) was comparable to Australian females aged 15 years and over (6.7% unemployed). We recruited a comparable proportion of women born overseas to the overall Australian population (31.5% vs. 26.7%), while a smaller proportion of our cohort lived in rural/remote areas (17.7% compared to 29.0%).

### 3.2. Reproductive Health, Family Planning and Genetic Factors

Approximately 90% of women overall were not aware of a reproductive life plan and approximately ~30% reported having a previous pregnancy. Thirty percent of active planners (the participant or their partner) reported currently or previously undertaking fertility treatment compared to 4.1% of non-active planners (*p* < 0.001). A subanalysis showed that those who reported use of fertility treatment were of a similar age (31.7 (4.0) vs. 30.6 (4.3) years *p* = 0.81) with a higher median BMI (25.7 (20.5, 30.9) vs. 23.3 (20.4, 26.2) kg/m^2^, *p* < 0.05) compared to those not reporting fertility treatment and a higher proportion were overweight or obese women (58.1% vs. 35.1%, *p* < 0.05). All other outcomes for reproductive health, family planning and genetic factors are displayed in Table 2.

### 3.3. General Physical Health, Medical Screening and Immunisation Status

General physical health characteristics, uptake of medical screening, routine health checks and testing and immunisation status are presented in Table 3, with no significant differences found between active and non-active planners. Approximately 17.0% of women over 25 years had never undertaken cervical screening for cancer prevention. A subanalysis comparing women those who had commenced screening (n = 159) with those who had not (n = 33), found no significant differences in demographic characteristics, with the exception of SEIFA classification, which indicated they were more likely to reside in an area of higher-level disadvantage (25.0% vs. 5.2%, *p* = 0.002). Twelve (n = 12) of those unscreened and aged over 25 years reported they had sought medical/health advice to prepare for pregnancy, and 6.1% had been tested for an STI within six months.

### 3.4. Actions to Prepare for Pregnancy, Unhealthy Lifestyle Behaviours and Modifiable Risk Factors

Women actively planning for pregnancy were more likely to report folic acid (75.2% vs. 30.6%, *p* < 0.001), iodine (29.8% vs. 16.2%, *p* = 0.01), pre-pregnancy (44.6% vs. 17.9%, *p* < 0.001) and/or vitamin D supplementation (38.9% vs. 22.5%, *p* = 0.003) compared with non-active planners (Table 4).

Overall, 6.6% were currently smoking, 85.3% had consumed alcohol in the previous three months and 59.0% indicated they had engaged in excessive drinking within the previous three months. Active planners were significantly more likely to report alcohol cessation in the previous three months in preparation for pregnancy, compared with women not actively planning (19.8% vs. 5.3%, *p* = 0.01). Yet, overall, active planners reported a similar median number of alcoholic drinks per week (4.0 (1.0, 7.0) vs. 3.0 (0.5, 5.5)) compared with women not actively planning (*p* = 0.35).

Approximately half of all women (54.4%) reported gaining weight in the previous 12 months, with 50.5% of these women reporting an increase of 3–5kg or more, and 19.1% reporting an increase of 6kg or more. Weighing habits, weight gain in previous 12 months, amount of weight gain and weight-related actions between the two groups did not differ significantly.

### 3.5. Subanalysis of Weight Behaviour and Weight Gain

Most women who reported weight gain in the previous 12 months were a healthy BMI (57.1%), while 21.0% were overweight, 21.0% were obese and 1.0% were underweight. The majority of those who reported a weight gain of 6kg or more within 12 months were obese (65.0%) or overweight (20.0%), with 15.0% a healthy BMI. Smaller weight increases of 1–2kg and 3–5kg in the previous year were most prevalent in women of a healthy BMI (81.3% and 58.5%, respectively), followed by those overweight (12.5% and 3.1%, respectively) and obese (26.4 and 15.1%, respectively).

Seventy-six percent of women who reported that they were currently trying to maintain a healthy weight were a healthy BMI, while 6.7% were underweight, 15.6% were overweight and 2.2% were obese. Of these, 38.9% reported gaining weight in the previous 12 months, while 52.2% had not and 8.9% were unsure.

The majority of women trying to lose weight reported weight gain in the previous 12 months (68.9%) and were an unhealthy BMI (34.4% obese, 25.8% overweight and 1.1% underweight) compared with 38.7% who were a healthy BMI. Most of those who reported no attempt to maintain nor lose weight were a healthy BMI (70.0% vs. 30.0% overweight/obese). Sixty percent of all women taking no weight-related actions reported weight gain within the previous 12 months. 

## 4. Discussion

We report that less than half of women planning a pregnancy had sought medical or health advice in preparation for pregnancy, and, of those that did, missed opportunities for important components of PCC existed. While uptake of cervical screening and up-to-date immunisation was relatively high, there was no difference between groups, with one in five active planners indicating they have never completed cervical screening and similar or greater proportions not immunised adequately for pregnancy. The intention to cease modifiable high-risk behaviours such as cigarette smoking and alcohol consumption was greater in those actively planning, yet the incidence and frequency of associated behaviours did not vary from those not actively planning a pregnancy. We also report increased weight gain in our cohort, emphasising the burden of accelerated progression to obesity in young, reproductive-aged women. Overall, despite some favourable areas of preconception health and pregnancy planning, including supplementation use, our results highlight several areas of preconception health warranting improved awareness, support and resources for women planning a pregnancy.

Our results show that less than half of women planning a pregnancy had sought health or medical advice as part of their pregnancy planning behaviour. This could be reflective of previous research demonstrating that women in preconception do not view themselves as a distinct group in need of healthcare [30,31]. It is plausible that women may plan for pregnancy individually, only engaging with their primary healthcare provider if difficulty in conceiving occurs [32]. The vast majority of active planners reported efforts to improve diet and increase exercise and had commenced preconception supplementation in line with national recommendations and consistent with previous Australian studies [19,33,34]. Our results indicate increased confidence in adopting self-managed behaviours and potentially lower awareness of aspects of PCC warranting health professional engagement [32,35], including cervical screening, immunisation and genetic testing, emphasising the need for targeted preconception health promotion in these areas in women planning a pregnancy.

Here, we found that approximately 1 in 5 eligible women had not commenced cervical screening. Interestingly, ~40.0% of these had sought medical/health advice to prepare for pregnancy, presenting a missed opportunity to initiate screening. Barriers for cervical screening are most commonly related to embarrassment, not acting on an intention to be screened, fear of pain and fear of results [36]. It is possible that women who had reported seeking advice and had not previously had a cervical screening test were provided with information but did not ultimately complete screening. Here, we found those that had not commenced screening were more likely to reside in areas of higher-level disadvantage compared with those who had performed screening. Given that engagement with a healthcare provider did not vary by screening status, this is likely not reflective of reduced access to healthcare or resources, with other contributory factors not captured by our survey potentially explaining results found. Similarly, 1 in 5 women or above were not adequately immunised for pregnancy and 50.0% of those with a known family history of a genetic condition had not been screened. We also report that approximately 30.0% of our cohort had undertaken an STI test within six months, in line with previous research [37]. Given uptake of all of screening types was similar in both groups, there is opportunity to improve these preventive health measures in primary care settings in all women preconception, irrespective of pregnancy planning. Barriers to PCC reported by GPs include lack of awareness of pregnancy intention and lack of presentation during preconception [38], mirroring results found here. Taken together, results concur with previous research highlighting the need to develop strategies that both encourage women into self-directed partnerships with their primary healthcare provider whilst also addressing barriers health providers experience for enhanced health communication overall [23].

Our findings indicate that women planning a pregnancy are not adhering to recommendations advising abstinence of alcohol during preconception [8], with just 11.5% reporting that they had stopped drinking, comparable to previous findings [39]. We found average weekly alcohol intake was comparable irrespective of planning status, potentially indicating complacency and/or ambivalence towards drinking in women actively planning for pregnancy. This could be due to the influence of individual and social factors as well as the normalisation of alcohol intake in women presenting as facilitators [18]. This highlights the need for strategies that encourage cessation of alcohol consumption when actively attempting to conceive given the discrepancy between intention and behaviour reported here. This could include leveraging off recent legislative changes on the introduction of warning labels on alcoholic beverages in Australia [40] and elsewhere [41].

Achieving and maintaining a healthy weight is highlighted as crucial in international evidence-based guidelines for preconception, pregnancy and post-pregnancy health [9]. Here, we report that ~55.0% of our cohort reported weight gain in the last 12 months, with 69.5% of these reporting an increase of three or more kilograms within the previous year. Even modest weight gain increases cardiovascular and chronic disease risk [42], whilst simultaneously contributing to obesity risk and conversion to higher BMI categories. Further, pregnancy is a recognised high-risk window for excessive gestational weight gain and postpartum weight retention [43,44]. This reaffirms the critical need for early intervention, prior to pregnancy, for weight gain prevention and/or weight loss where required, given the associated pregnancy and future health risk for mother and child [45]. Overall, approximately half of all women were currently trying to lose weight, and, encouragingly, the majority of active planners reported they had improved their diet and exercise behaviours to prepare for pregnancy, potentially reflective of enhanced motivation to ensure optimised health in pregnancy, as previously reported [46].

Reproductive and fertility behaviours varied, potentially in line with women’s pregnancy planning intentions. Women not actively planning a pregnancy were more likely to report hormonal and barrier contraceptive use, which is positive, both decreasing the likelihood of an unplanned pregnancy and enabling opportunities for PCC. Interestingly, one quarter of non-active planners reported using the withdrawal method regularly, potentially indicating complacency and/or lack of awareness, given this is recognised as one of the least effective forms of contraception with a ~20.0% failure rate [47,48]. Conversely, current or previous assisted fertility treatment was reported by a higher proportion of women actively planning for pregnancy compared to non-active planners. There was no difference in age in those who had engaged in fertility treatment compared with those who had not; however, there were higher proportions of overweight or obesity overall. One in six Australian couples experience fertility problems [49], in line with international estimates [50]. Most fertility treatment in Australia is provided in the private health system, and the high rate of fertility treatment reported by active planners in this study may reflect increased likelihood of commencing private health insurance to access treatment (as reported previously) as well as the overall higher socioeconomic status (SES) of participants [51]. Fertility treatment reported in non-active planners is likely reflective of previous treatment; however, this cannot be delineated from current use due to the survey structure. Similarly, we were unable to explore causes of infertility and associated treatment overall.

### Strengths and Limitations

Our rigorously developed questionnaire assessed an extensive range of health and lifestyle behaviours in accordance with the majority of national PCC recommendations [21]. Our stratification by stage of pregnancy planning strengthens the understanding of PCC uptake by enabling differences in behaviour to be observed. While the cross-sectional design of our study can explore associations, we are unable to confirm causal relationships and inferential statistics were not possible.

Our cohort consisted of women who had private health insurance, which may limit the generalisability of our results to other populations owing to an overall higher socio-demographic profile. The private health system uniquely provides an opportunity to explore preconception behaviours before conception due to waiting periods, of typically 12-months, before hospital-based healthcare claims can be made. Whilst this study group are of a higher SES, ~50.0% of Australian women of reproductive age have private health insurance and 26.0% birth in private hospitals [13]; hence, our results are relevant to a significant proportion of Australian women, further emphasized by comparability with Australian population census data.

## 5. Conclusions

Our results demonstrate significant divergence from PCC recommendations in women planning for pregnancy, with several areas of preconception health that warrant improved health promotion. While women appear to be more receptive to self-managed aspects of preconception health, including supplementation, weight management and intention to cease higher-risk behaviours including alcohol intake, we found minimal variation in behaviour compared with women not actively planning a pregnancy. Given the potential for evidence-based PCC to optimise fertility and reduce adverse maternal and child outcomes, efforts are required to improve PCC knowledge, awareness and uptake through strengthened partnerships between women and their healthcare providers. Future research would benefit from extension into other populations as well as prospective studies examining causal associations between preconception health behaviours and associated outcomes during pregnancy.

## Figures and Tables

**Table 1:** Participant characteristics stratified by pregnancy intention.

Characteristic	All (n = 294)	Active Planners (n = 121)	Non-Active Planners (n = 173)	*p*-Value
**Age (years) Mean (SD)**	***n = 195***	***n = 89***	***n = 106***	
	30.7 (4.3)	31.4 (4.4)	30.2 (4.1)	0.35
**BMI (kg/m^2^) Median (IQR)**	***n = 193***	***n = 88***	***n = 105***	
	23.7 (20.1, 26.8)	24.2 (20.8, 27.7)	23.1 (20.3, 26.0)	0.05
**Country of birth**	***n = 197***	***n = 90***	***n = 107***	
Australia	135 (68.5%)	56 (62.2%)	79 (73.8%)	0.08
Outside Australia	62 (31.5%)	34 (37.8%)	28 (26.2%)	
**Education**	***n = 196***	***n = 89***	***n = 107***	
School Only	10 (5.1%)	6 (6.7%)	4 (3.7%)	0.18
Certificate/Diploma/Apprenticeship	69 (35.2%)	36 (40.5%)	33 (30.8%)	
University	117 (59.7%)	47 (52.8%)	70 (65.4%)	
**Employment**	***n = 196***	***n = 89***	***n = 107***	
Employed	184 (93.9%)	86 (96.6%)	98 (91.6%)	0.14
Unemployed	12 (6.1%)	3 (3.4%)	9 (8.4%)	
**Area of residence**	***n = 192***	***n = 87***	***n = 105***	
Urban	158 (82.3%)	73 (83.9%)	20 (19.0%)	0.29
Rural/remote	33 (17.7%)	14 (16.1%)	85 (81.0%)	
**SEFIA**	***n = 191***	***n = 86***	***n = 105***	
Higher-level disadvantage	18 (9.4%)	8 (9.3%)	10 (9.5%)	0.98
Moderate-level disadvantage	59 (30.9%)	26 (30.2%)	33 (31.4%)	
Lower-level disadvantage	114 (59.7%)	52 (60.5%)	62 (59.0%)	
**Annual household income (AUD)**	***n = 196***	***n = 89***	***n = 107***	
<$40,000	8 (4.1%)	4 (4.5%)	4 (3.7%)	0.74
$41,000–$64,999	17 (8.7%)	9 (10.1%)	8 (7.5%)	
$65,000–$80,000	24 (12.2%)	8 (9.0%)	16 (15.0%)	
>$81,000	135 (68.9%)	62 (69.7%)	73 (68.2%)	
Prefer not to answer	12 (6.1%)	6 (6.7%)	6 (5.6%)	
**Relationship Status**	***n = 196***	***n = 89***	***n = 107***	
Married/De facto	184 (93.9%)	87 (97.8%)	97 (90.7%)	0.04
Unmarried	12 (6.1%)	2 (2.3%)	10 (9.3%)	

Total *n* for each variable may vary based on the total number of responses.

**Table 2:** Reproductive health, actions and awareness, stratified by pregnancy intention.

Factor or Action	All (n = 294)	Active Planners (n = 121)	Non-Active Planners (n = 173)	*p*-Value
**Awareness of reproductive life plan**	***n = 255***	***n = 110***	***n = 145***	
Yes	25 (9.8%)	10 (9.1%)	15 (10.3%)	0.74
**Previous pregnancy**	***n = 57***	***n = 31***	***n = 26***	
Yes	20 (35.1%)	12 (38.7%)	8 (30.8%)	0.53
**Regular contraception choice**				
No contraception	***n = 232***	***n = 103***	***n = 129***	<0.001
	87 (37.5%)	65 (63.1%)	22 (17.1%)	
Withdrawal	***n = 213***	***n = 89***	***n = 124***	<0.01
	41 (19.2%)	9 (10.1%)	32 (25.8%)	
Barrier	***n = 223***	***n = 92***	***n = 131***	<0.001
	62 (27.8%)	14 (15.2%)	48 (36.6%)	
Hormonal	***n = 233***	***n = 95***	***n = 138***	<0.001
	85 (25.8%)	12 (12.6%)	73 (52.9%)	
**Fertility treatment (previous or current treatment of participant or their partner)**	***n = 255***	***n = 110***	***n = 145***	
Yes	40 (15.7%)	34 (30.9%)	6 (4.1%)	<0.001
**Personal/family history of genetic condition**	***n = 199***	***n = 90***	***n = 109***	
Yes	40 (20.1%)	19 (21.1%)	21 (19.3%)	0.95
No	119 (59.8%)	53 (58.9%)	66 (60.6%)	
Unsure	40 (20.1%)	18 (20.0%)	22 (20.2%)	
**Tested for genetic conditions**	***n = 40***	***n = 19***	***n = 21***	
Yes	23 (57.5%)	10 (53.6%)	13 (61.9%)	0.84
No	15 (37.5%)	8 (42.1%)	7 (33.3%)	
Unsure	2 (5.0%)	1 (5.3%)	1 (4.8%)	

Total *n* for each variable may vary based on the total number of responses.

**Table 3:** General physical health and screening, stratified by pregnancy intention.

Factor or Action	All (n = 294)	Active Planners (n = 121)	Non-Active Planners (n = 173)	*p*-Value
**BMI category**	***n = 193***	***n = 88***	***n = 105***	
Underweight	7 (3.6%)	4 (4.5%)	3 (2.9%)	0.43
Healthy	111 (57.5%)	45 (51.1%)	66 (62.9%)	
Overweight	39 (20.2%)	21 (23.9%)	19 (18.1%)	
Obese	36 (18.7%)	18 (20.5%)	17 (16.2%)	
**Undertaken cervical screening/pap smear**	***n = 197***	***n = 90***	***n = 107***	
Yes	159 (80.7%)	73 (81.1%)	86 (80.4%)	0.97
No (aged, >25yrs)	33 (16.8%)	15 (16.7%)	18 (16.8%)	
No (aged, ≤25yrs)	5 (2.5%)	2 (2.2%)	3 (2.8%)	
**STI test (within 6 months)**	***n = 197***	***n = 90***	***n = 107***	
Yes	57 (28.9%)	31 (34.4%)	26 (24.3%)	0.12
**Dental Check Up (within 12 months)**	***n = 252***	***n = 110***	***n = 142***	
Yes	181, (71.8%)	81, (73.6%)	100, (70.4%)	0.06
**Currently experiencing gum/teeth problem**	***n = 252***	***n = 110***	***n = 142***	
Yes	32 (12.7%)	15 (13.6%)	17 (12.0%)	0.69
**Up-to-date immunisation**	***n = 197***	***n = 90***	***n = 107***	
Measles Mumps Rubella (MMR)	152 (77.2%)	72 (80.0%)	80 (74.8%)	0.38
Hepatitis B	139 (70.6%)	65 (72.2%)	74 (69.2%)	0.64
Chicken Pox (Varicella)	124 (62.9%)	55 (61.1%)	69 (59.8%)	0.63
Tetanus/Diphtheria/Pertussis (whooping cough)	156 (79.2%)	71 (78.9%)	85 (79.4%)	0.92
Influenza	101 (51.3%)	46 (51.1%)	55 (51.4%)	0.97
None of the above	18 (9.1%)	7 (7.8%)	11 (10.3%)	0.54
Unsure	4 (2.0%)	1 (1.1%)	3 (2.8%)	0.40

Total *n* for each variable may vary based on the total number of responses.

**Table 4:** Actions to prepare for pregnancy, unhealthy lifestyle behaviours and modifiable risk factors, stratified by pregnancy intention.

Factor or Action	All (n = 294)	Active Planners (n = 121)	Non-active Planners (n = 173)	*p*-Value
**Current actions to prepare for pregnancy**	***n = 294***	***n = 121***	***n = 173***	
*Supplement use:*				
Taking folic acid	144 (49.0%)	91 (75.2%)	53 (30.6%)	<0.001
Taking iodine	64 (21.8%)	36 (29.8%)	28 (16.2%)	0.01
Taking a pre-pregnancy supplement	85 (28.9%)	54 (44.6%)	31 (17.9%)	<0.001
Taking folic acid/iodine/pre-pregnancy supplement *	155 (52.7%)	98 (81.0%)	57 (33.0%)	<0.001
Taking vitamin D	86 (29.3%)	47 (38.9%)	39 (22.5%)	<0.01
Taking other supplements	40 (13.6%)	14 (11.6%)	26 (15.0%)	0.40
*Diet:*				
Improving diet	190 (64.6%)	77 (63.6%)	113 (65.3%)	0.77
*Physical activity:*				
Increasing exercise	176 (59.9%)	67 (55.4%)	109 (63.0%)	0.19
*Psychosocial:*				
Improving sleeping patterns/decreasing stress	78 (26.5%)	27 (22.3%)	51 (29.5%)	0.17
*Healthcare:*				
Seeking medical/health advice	119 (40.5%)	57 (47.1%)	62 (35.8%)	0.05
*Other:*				
Trying to stop/decrease smoking	18 (6.1%)	7 (5.8%)	11 (6.4%)	0.84
Trying to stop/decrease drinking alcohol	74 (25.2%)	43 (35.5%)	31 (17.9%)	<0.01
Not doing any of the above	26 (8.8%)	5 (4.1%)	21 (12.1%)	0.02
**Smoking status**	***n = 252***	***n = 110***	***n = 142***	
Yes, current smoker	17 (6.6%)	8 (7.3%)	9 (6.3%)	0.92
Never smoked/quit smoking	235 (91.8%)	100 (90.9%)	131 (92.3%)	
Prefer not to answer	4 (1.6%)	2 (1.8%)	2 (1.4%)	
**Consumed alcohol in previous 3 months**	***n = 225***	***n = 95***	***n = 130***	
Yes	192 (85.3%)	81 (85.3%)	111 (85.4%)	0.98
**Excessive drinking**	***n = 227***	***n = 96***	***n = 131***	
One or more times	134 (59.0%)	54 (56.3%)	80 (61.1%)	<0.01 **
Nil	41 (18.1%)	14 (14.6%)	27 (20.6%)	
Unsure	26 (11.5%)	9 (9.4%)	17 (13.0%)	
Stopped drinking for pregnancy	26 (11.5%)	19 (19.8%) **	7 (5.3%) **	
**Average alcoholic drinks per week in past 3 months. Median (IQR)**	***n = 225***	***n = 92***	***n = 133***	
	3.0 (0.0, 6.0)	4.0 (1.0, 7.0)	3.0 (0.5, 5.5)	0.35
**Recreational drug use**	***n = 248***	***n = 108***	***n = 140***	
Yes, within 1 month	13 (5.2%)	5 (4.6%)	8 (4.3%)	0.95
Yes, within 1 year	13 (5.2%)	7 (6.5%)	6 (4.3%)	
Yes, but not within 1 year	48 (19.4%)	21 (19.4%)	27 (19.3%)	
Never	169 (68.1%)	73 (67.6%)	96 (68.6%)	
Prefer not to answer	5 (2.0%)	2 (1.9%)	3 (2.1%)	
**Weighing habits**	***n = 193***	***n = 88***	***n = 105***	
Regular	93 (48.2%)	47 (53.4%)	46 (43.8%)	0.18
Irregular	100 (51.8%)	41 (46.6%)	59 (56.2%)	
**Weight gain in previous 12 months**	***n = 193***	***n = 88***	***n = 105***	
Yes	105 (54.4%)	43 (48.9%)	62 (59.1%)	0.37
No	74 (38.3%)	38 (43.2%)	36 (34.3%)	
Unsure	14 (7.3%)	7 (8.0%)	7 (6.7%)	
**Amount weight gain in previous 12 months**	***n = 105***	***n = 43***	***n = 62***	
1–2 kg	32 (30.5%)	14 (32.6%)	18 (29.0%)	0.79
3–5 kg	53 (50.5%)	20 (46.5%)	33 (53.2%)	
6 kg or more	20 (19.0%)	9 (20.9%)	11 (17.7%)	
**Weight related actions (trying to..)**	***n = 254***	***n = 110***	***n = 144***	
Maintaining a healthy weight	116 (45.7%)	53 (48.2%)	63 (43.8%)	0.73
Lose weight	125 (49.2%)	51 (46.4%)	74 (51.4%)	
Neither of the above	13 (5.1%)	6 (5.5%)	7 (4.9%)	

* Participants who selected one or more of the following options: (currently) taking folic acid/folate/Blackmores I-Folic/iodine/multivitamin for pre-pregnancy or (currently) taking other action (and listed pregnancy/pre-pregnancy multivitamin or similar). ** Significant difference (*p* = 0.001) identified here after Bonferroni correction. Total n for each variable may vary based on the total number of responses.

## Supplementary Materials

The following are available online at https://www.mdpi.com/2077-0383/9/6/1701/s1: Improving Health in Australia, Monash Pre-Pregnancy Questionnaire.
