# Supplementary material for: Preconception Health and Lifestyle Behaviours of Women Planning a Pregnancy: A Cross-Sectional Study
Source: J Clin Med. 2020 Jun 2;9(6):1701. doi: 10.3390/jcm9061701 (PMC7355494; doi:10.3390/jcm9061701)
Supplement: Supplementary file 1 [file jcm-09-01701-s001.pdf]

Supplementary Material S1 (Preconception Health and Lifestyle Behaviours of Women Planning a Pregnancy: A Cross Sectional Study)

Improving Health in Australia, Monash Pre-Pregnancy Questionnaire

1. **I agree to participate in this research study. I understand the purpose and nature of this study and I am participating voluntarily. I understand that I can withdraw from the study at any time, without any penalty or consequences.**
   - ☐ Yes
   - ☐ No <end survey>
2. **I grant permission for the data generated from this questionnaire to be used by Monash University to inform educational material and intervention design to improve the future health of mothers and their children.**
   - ☐ Yes
   - ☐ No <end survey>

Before you begin the questionnaire, it is important for us to know whether or not you are <u>currently pregnant</u>. The questions we ask will differ slightly depending on your circumstances.

3. **Are you currently pregnant? *(Please select <u>one</u> option only)***
   - ☐ Yes <redirect to complete our Pre-pregnancy Questionnaire_Version 5 (ii)_CURRENTLY pregnant>
   - ☐ No

1. Planning Around Future Pregnancy

The questions in this section ask for detail about reproductive and contraception information.

1. **Are you planning a pregnancy in future? *(Please select <u>one</u> option only)***
   - ☐ Yes, I am currently trying to conceive
   - ☐ Yes within 1 year
   - ☐ Yes within 5 years
   - ☐ Yes within 10 years *(Please proceed to question 3)*
   - ☐ No I have completed my family *(Please proceed to question 3)*
   - ☐ No I do not wish to have a future pregnancy *(Please proceed to question 3)*
   - ☐ I prefer not to answer *(Please proceed to question 3)*
   - ☐ I am unsure *(Please proceed to question 3)*

The question below asks about any actions you are currently taking to actively plan for a pregnancy.

2. **From the items below, what are you currently doing to improve your health in preparation for pregnancy? *(Please select <u>all</u> that you are currently doing)***

- ☐ taking folic acid
- ☐ taking iodine
- ☐ taking vitamin D
- ☐ trying to stop or cut down smoking
- ☐ trying to stop or cut down drinking alcohol
- ☐ Improving diet
- ☐ increasing exercise levels
- ☐ improving sleeping patterns
- ☐ seeking medical/health advice
- ☐ taking some other action, please describe ___________
- ☐ I am not doing any of the above currently

3. **Please tick to show if you have used any of the following methods of contraception in the 6 months when engaging in sexual intercourse.**
(Even if you stopped using contraception, you can still tick option b. *'Nothing now – I am trying to conceive'* and tick other methods you had previously used within the past 6 months) ***(Please select one response for each of the options a to n)***

	**How often in the past 6 months have you used the following contraception method when engaging in sexual intercourse?**			
**Type**	**Never** *0 in 10*	**Occasionally** *1-4 in 10*	**Some of the time** *5-9 in 10*	**Every time** *10 in 10*
a. Nothing	☐	☐	☐	☐
b. Nothing now – I am trying to conceive	☐	☐	☐	☐
c. Withdrawal	☐	☐	☐	☐
d. Condoms	☐	☐	☐	☐
e. Birth control pills (Oral contraceptives)	☐	☐	☐	☐
f. Cap/diaphragm	☐	☐	☐	☐
g. Contraceptive Injection (Depo-Provera)	☐	☐	☐	☐
h. Contraceptive Implant (Implanon)	☐	☐	☐	☐
i. Intrauterine device (IUD or coil)	☐	☐	☐	☐
j. Intrauterine system (IUS e.g. Mirena)	☐	☐	☐	☐
k. NuvaRing vaginal contraceptive ring	☐	☐	☐	☐
l. Abstinence (I am not having sex)	☐	☐	☐	☐
m. Emergency contraception (Morning-After	☐	☐	☐	☐
n. Other (please give details)	☐	☐	☐	☐

4. **Have you or your partner been treated or currently undergoing treatment for infertility?**

- ☐ Yes
- ☐ No

5. **Have you heard of a reproductive life plan? A reproductive life plan is a plan to have children (or not), the number, spacing and timing of intended children)?**

- ☐ Yes
- ☐ No

2. Lifestyle Information

The following section asks about your lifestyle, behaviours and experiences.

1. **In regards to your weight, are you currently:**

☐ A healthy weight (BMI: ≥18.5 - <25)
☐ Overweight (BMI: ≥25)
☐ Underweight (BMI: <18.5)
☐ Unsure

2. **Are you currently attempting to maintain a healthy weight or lose weight?**

☐ Yes, maintain a healthy weight
☐ Yes, lose weight
☐ No

3. **Are you currently experiencing any problems with your teeth or gums (e.g. tooth decay or holes in your teeth, bleeding gums, periodontal or gum disease)?**

☐ Yes
☐ No

4. **Have you visited the dentist in the past 12 months?**

☐ Yes
☐ No (If no, please specify why________________________)

5. **Have you ever smoked?**

☐ Yes
☐ No
☐ Prefer not to answer

6. **If *yes*, are you currently smoking?**

☐ Yes, I am smoking _ _ _ cigarettes a day and I am currently not trying to quit.
☐ Yes, I am smoking _ _ _ cigarettes a day, but I am currently trying to quit in case I become pregnant.
☐ Yes, I am smoking _ _ _ cigarettes a day, but I am currently trying to quit for other reasons.
☐ No, I am not currently smoking in case I become pregnant.
☐ No, I am not currently smoking for other reasons.

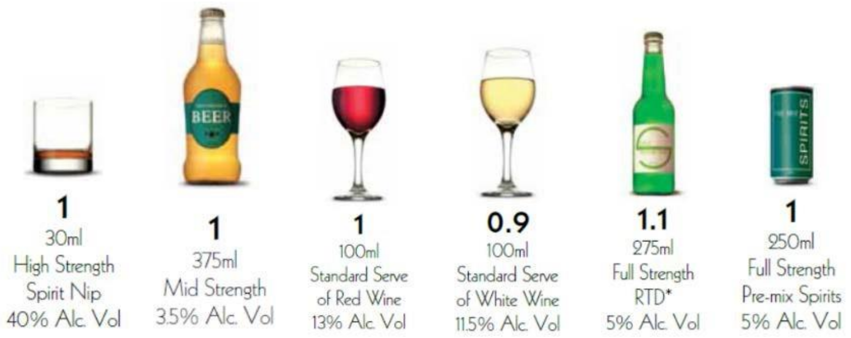

Image: Examples of a standard drink of alcohol Adapted from Australian Government National Health and Medical Research Council (NHMRC) 2009, Australian guidelines to reduce health risks from drinking alcohol https://www.nhmrc.gov.au/guidelines-publications/ds10

7. **On average, how many standard drinks of alcohol did you have per week in the past 3 months?** *(Please refer to the image above for examples of a standard drink of alcohol. If you are not drinking alcohol please put 0)*

	**Monday - Thursday**	**Weekends (Friday to Sunday)**
Average drinks consumed in the past 3 months	– – –	– – –

8. **In the past 3 months, how many times did you consume four standard drinks or more on a single occasion?** *(Please refer to the image above for examples of a standard drink of alcohol. If you are not drinking alcohol please put 0)*

☐ Number of times (please enter number of times) ___________
☐ I don't remember/I don't know
☐ I had stopped drinking alcohol because I was trying to get pregnant

9. **Have you ever taken recreational drugs (e.g. Cocaine/crack, Marijuana, Methamphetamines, Methadone, Heroin, Ecstasy)?**

☐ Yes
☐ No ***(Please go to*** section 3: Pre-pregnancy health information received***)***
☐ Prefer not to answer ***(Please go to*** section 3: Pre-pregnancy healthy information received)

10. **If *yes*, when was the last time you took recreational drugs?** ***(Please select all that apply)***

☐ Today
☐ Last week
☐ Last month
☐ Last year
☐ 5+ years ago

☐ I have stopped taking recreational drugs in case I became pregnant
☐ I have stopped taking recreational drugs for other reasons
☐ Prefer not to answer

3. Medical History and Previous Pregnancies

1. **Do you have a personal or family history of any genetic conditions?**

☐ Yes
☐ No
☐ Unsure

2. **If *yes*, were you tested for any of these genetic conditions?**

☐ Yes
☐ No
☐ Unsure

3. **Please tick any vitamins or supplements you are currently taking.** ***(Please select all that apply)***

Vitamin or supplement name	Currently taking (please tick)
Multivitamin for pre-pregnancy (e.g. Blackmores Conceive Well Gold)	☐
Blackmores I-Folic	☐
Folate	☐
Iron	☐
Iodine	☐
Vitamin D	☐
Other (please specify) ____________________	☐
None	☐

4. **Are you up to date with any of the following immunisations?** ***(Please select all that apply)***

☐ Measles, Mumps, Rubella (MMR)
☐ Hepatitis B
☐ Chickenpox (Varicella)
☐ Tetanus/Diphtheria/Pertussis (whooping cough booster)
☐ Influenza
☐ Others *(Please specify)* ____
☐ None of the above

5. **Have you undertaken a screening for cervical cancer prevention (e.g. Pap smear)?**

☐ Yes
☐ No

6. **Have you been tested for any sexually transmitted infections (e.g. Gonorrhoea, chlamydia) in the last 6 months?**

☐ Yes
☐ No

7. **Have you been pregnant before?**

☐ Yes
☐ No

4. Information About You

The following questions relate to your individual details and characteristics

1. **What is your date of birth?**

   _ _/_ _/_ _ _ _

2. **What country where you born in?**

☐ Australia
☐ United Kingdom
☐ India
☐ Sri Lanka
☐ New Zealand
☐ Afghanistan
☐ Philippines
☐ Mauritius
☐ China
☐ South Africa
☐ Other (please specify) ____________________

3. **What is your postcode?**

   _ _ _ _

4. **Do you identify as being Aboriginal and/or Torres Strait Islander?** ***(Please select one option only)***

☐ Yes, Aboriginal
☐ Yes, Torres Strait Islander
☐ Yes, Aboriginal and Torres Strait Islander
☐ No, neither

5. **What is the highest level of schooling you have completed?** ***(Please select one option only)***

☐ Year 9 or below (Junior High)
☐ Year 10 or equivalent (Middle High)
☐ Year 11 or equivalent
☐ Year 12 or equivalent (senior high)

6. **What is the highest post-school qualification you have completed? (please select one option only)**

☐ No post-school qualification/s
☐ Certificate
☐ Diploma
☐ Bachelor degree and above
☐ Apprenticeship

7. **Which of the following best describes your <u>main current</u> employment status?**

☐ In full time paid work
☐ In part time or casual paid work
☐ Work without pay (e.g. in a family business)
☐ Home duties only – no paid work
☐ Studying
☐ Unemployed – looking for work
☐ Unpaid voluntary work
☐ Unable to work due to sickness or injury
☐ Other *(Please specify)* _

8. **What is the average gross income (before tax) that your household receives each year, including pensions, allowances, and financial support?** ***Please note that 'household' refers to the total of all major income earners.***

☐ $40,000 or less
☐ $41,000 – 64,999
☐ $65,000 – 80,000
☐ More than $81,000
☐ I prefer not to answer

9. **What is your current marital status?**

- ☐ Married
- ☐ Defacto
- ☐ Separated
- ☐ Divorced
- ☐ Widowed
- ☐ Never married

10. What is your height without shoes? This is important, ***please measure your height or provide the closest estimate in centimeters or meters or feet.***

____

11. What is your weight without shoes? This is important, ***please measure your current weight or provide the closest estimate in kg.***

______________ kg

12. How often do you weigh yourself?

- ☐ Daily
- ☐ Weekly
- ☐ Monthly
- ☐ Occasionally
- ☐ Never

13. Have you gained weight in the past year?

- ☐ Yes
- ☐ No
- ☐ Unsure

14. If yes, how much weight have you gained?

- ☐ 1 – 2 kg
- ☐ 3 – 4 kg
- ☐ 5 kg
- ☐ 6 – 10 kg
- ☐ Greater than 10 kg
